# Evolution of endoscopic surgery in the treatment of inverted papilloma

**DOI:** 10.5935/1808-8694.20130003

**Published:** 2015-10-14

**Authors:** Fábio de Azevedo Caparroz, Luciano Lobato Gregório, Eduardo Macoto Kosugi

**Affiliations:** 3^rd^ year ENT resident physician; 3^rd^ year ENT resident physician; MSc and PhD in Otorhinolaryngology; Chief preceptor of the residency program and coordinator of the Fellowship Program in Rhinology - Department of Otorhinolaryngology and Head and Neck Surgery - UNIFESP/EPM). Department of Otorhinolaryngology and Head and Neck Surgery - Paulista School of Medicine - Federal University of São Paulo (UNIFESP)

**Keywords:** papilloma, inverted, paranasal sinus neoplasms, endoscopic sinus surgery

## Abstract

Inverted papilloma (IP) has several treatment avenues. The endoscopic approach in the last decade has proven to be a good option over the traditional approach.

**Objective:**

Describe the epidemiological profile of patients with inverted Papilloma, describe our experience on managing this tumor and compare our data with the literature. Study Design: Cross-sectional, historical cohort.

**Method:**

Retrospective study of medical records of 17 patients treated for histopathologically-confirmed inverted papilloma between 2005 and 2011. We assessed patients age, gender, tumor side, symptoms, diagnosis, comorbidities and habits, Krouse staging, surgical approach, intraoperative and postoperative, and malignant postoperative recurrence and also the correlation between recurrence with preoperative staging, the surgical approach used, and the presence of malignancy.

**Results:**

Five (29.41%) patients were classified as Krouse stage T2, 9 (52.94%) as T3 and 3 (17.65%) as T4. Three (17.65%) patients had malignancy and the recurrence rate was 23.5% (4 pacients). Eleven patients (64.70%) underwent endoscopic approach, 3 (17.6%) the combined aprroach (endoscopic assisted) and 3 (17.6%) external approach.

**Conclusion:**

The endoscopic approach is currently becoming a method not only effective but also safe for the treatment of more advanced stages of IP.

## INTRODUCTION

Inverted papilloma (IP) is a common benign tumor of the nose and paranasal sinuses, originating from the Schneiderian epithelium of the lateral nasal wall, first described in 1854 by Ward^1^. They make up approximately 0.5% to 4% of the primary nasal tumors, and are more common on the fifth and sixth decades of life, having an incidence of 0.5 to 1.5 cases per 100,000 inhabitants. Its main characteristics are a tendency to recurrence, local aggressiveness and association with malignancy^2^. They may invade the lacrimal system, the orbit, the intracranial cavity and cause bone erosion and destruction and that of adjacent soft tissue^3^. The malignization rates in the literature vary between 5% and 15%^4^.

Although the clinical progress is well documented in the literature, the etiology of the inverted papilloma is uncertain. Recent studies have shown that besides having a 33% association with the human papilloma virus (HPV)^5^, there is an increase in the presence of HPV types 16 and 18 in tumors with severe dysplasia, or even carcinoma^6^. These results show that, maybe, the HPV has an important role to play in the etiology of inverted papilloma^7^.

Diagnosis may be suspected by means of the medical interview, physical exam and image exam - such as CT scan and MRI, but it can only be confirmed by means of histology. Krouse^3^^,^^8^^,^^9^, and later on, Han et al.^4^^,^^8^ proposed classifications which enable comparisons between surgical techniques according to tumor staging. The standard treatment initially proposed to resect the inverted papilloma was the external access by lateral rhinostomy, described by Moure, in 1902^9^. Notwithstanding, this technique has complications, both temporary and permanent, such as atrophic rhinitis, persistent crusts and purulent rhinorrhea, let alone the scar.

In recent years, we have seen the progress in the endoscopic-assisted resection of inverted papillomas with lower rates of recurrence in relation to the beginning of the era of endoscopy^9^. Moreover, the endoscopic resection is done in a minimum, with the same effectiveness to control the tumor, thus avoiding the comorbidities of the conventional approach^8^. The development of the endoscope brought about a number of advantages when compared to external approaches, such as better view of the operation field, less resection of viable tissue not involved by the tumor - causing less crust formation, less bleeding and less postoperative pain, in other words, reducing morbidity and maintaining the same effectiveness^4^^,^^9^. Today, there is a trend of considering the endoscopic technique alone as the gold standard to treat most cases of inverted papillomas, even in the most advanced stages^10^, including the treatment of recurrences^11^.

The goal of our study was to discuss a sample of patients seen in our department, with a diagnosis of inverted papilloma, confirmed by histopathology, and describe our experience in its treatment, comparing the results with reports in the national and international literature.

## METHOD

Cross-sectional historical cohort study, assessing patients seen in a rhinology department with a pathology report of inverted papilloma during the period of January of 2005 and October of 2011. This study project has been submitted to and approved by the Ethics Committee in Research of our Institution, under protocol number # 2079/11. Inclusion criteria for the sample were a confirmed histological diagnosis in the surgical specimen and minimum follow up time of 4 months. From an initial sample of 26 patients, we included 17 with an average follow up time of 18.3 months and we took off the patients with follow up time of less than 4 months (loss of follow up). All the patients were classified according to the classification proposed by Krouse^3^, based on the CT scan of preoperative paranasal sinuses.

We assessed: age, gender, tumor side, symptoms upon diagnosis (nasal obstruction, anterior or posterior rhinorrhea, epistaxis, hyposmia, cacosmia, headache and facial pain), comorbidities and habits, Krouse staging, surgical approach, intra and postoperative complications, malignization and postoperative recurrence. We also assessed the possibility of correlation between the recurrences with higher preoperative staging, with the surgical approach utilized and the presence of malignization utilizing the Pearson's coefficient. All the patients were submitted to surgery by the same team, by external approach, endonasal endoscopic or a combined approach. The postoperative follow up was carried out by nasal endoscopy in series, weekly in the first month and monthly until the sixth month. Later on, the follow up was every six months. In suspecting clinical recurrence, we carried out a CT scan of the paranasal sinuses.

## RESULTS

The study included 17 patients, seen between January of 2005 and October of 2011. The sample characteristics are seen on Table 1.

**Table 1:** Sample characteristics.

Data		N	%
Gender	Males	14	82.3
	Females	3	17.6
Side	Left	8	47.0
	Right	9	52.9
	Mean	58.76	–
	SD	7.42	–
Age (years)	Minimum	47	–
	Median	58	–
	Maximum	73	–

N: Number; %: percentage.

The symptoms presented by the patients are described on Table 2.

**Table 2:** Symptom distribution.

Symptom	N	%
Nasal obstruction	17	100
Epistaxis	10	58.8
Hyposmia	9	52.9
Anterior rhinorrhea	7	41.1
Headache	6	35.2
Cough	4	23.5
Posterior rhinorrhea	2	11.7
Cacosmia	2	11.7
Facial pain	1	5.8

N: Number; %: percentage.

The comorbidities of the patients are listed on Table 3.

**Table 3:** Comorbidities.

Comorbidity	N	%
Smoking	8	47.0
Hypertension	8	47.0
Alcohol drinking	2	11.7
Coronary failure	2	11.7
Diabetes	1	5.8
Dyslipidemia	1	5.8

N: Number; %: percentage.

The patients were classified according to Krouse's staging system. No patient was classified in stage I; five patients (29.41%) in stage II; nine patients (52.94%) in stage III; and three patients (17.65%) in stage IV.

Complications and malignization found are described on Table 4.

**Table 4:** Complications and malignization.

Characteristics		Number	%
Intraoperative complication		0	0
Postoperative complication	Synechia	1	5.8
	Septal perforation	1	5.8
	Oroantral fstula	1	5.8
Malignization		3	17.6

%: percentage.

There were four recurrences in our sample, corresponding to 23.5% of the cases. There were no correlations between the recurrences and a higher staging, through the endoscopic approach only, or malignization. The distribution of recurrences according to staging is shown on Table 5. Despite the trend shown on the table displaying the increase in recurrence with worsening of the staging, there were no associations between these two variables (Pearson r = 0.05).

**Table 5:** Distribution of stages and recurrences.

Stage	Krouse's stage		Recurrence	
	N	(% of cases)	N	(% of the stage)
I	0	–	0	–
II	5	29.4	1	20
III	9	52.9	2	22.2
IV	3	17.6	1	33.3
Total	17	100	4	23.5

N: Number; %: percentage.

The assessment of recurrences according to the approach and staging is shown on Table 6. Moreover, we can see the number of cases and the approach (external, combined or endoscopic) to which the patient was submitted to. In 64% of the cases, the approach was the endoscopic alone. There were no correlations between the recurrences and the approach (r = 0.1) or with malignancy (r = 0.1).

**Table 6:** Recurrence cases per approach and stage.

Krouse's staging															
Approach	I				II			III			IV			Total	
	Case	Recur	%	Case	Recur	%	Case	Recur	%	Case	Recur	%	Case	Recur	%
External	–	–	–	–	–	–	1	0	0	2	1	50	3	1	33.3
Combined	–	–	–	–	–	–	2	1	50	1	0	0	3	1	33.
Endoscopic	–	–	–	5	1	20	6	1	16.6	–	–	–	11	2	18.1
Total	–	–	–	5	1	20	9	2	22.2	3	1	33.3	17	4	23.5

Recur: Recurrence; %: percentage of recurrences per approach in each group.

## DISCUSSION

Sinonasal inverted papilloma is a benign neoplasia which can be diagnosed at any age, predominating between the fifth and sixth decades of life, and specially among males^12^, and such information was confirmed in our study - mean age of 58 years and a predominance of males (82.3%). The most commonly found symptoms were unilateral nasal obstruction (100% of the cases), epistaxis (58.8%), hyposmia and/or anosmia (52.9%), anterior rhinorrhea (41.1%) and headaches (35.3%). There was a greater incidence of anterior epistaxis (58.8%) upon diagnosis, vis-à-vis the literature, which varies between 6%^13^ and 20%^14^, and this is a differential symptom in relation to rhinosinusitis. Even then, the low specificity of symptoms makes it difficult to distinguish inverted papillomas from other sinonasal tumors and it delays the diagnosis in many cases. Besides epistaxis, it is worth remembering that most signs and symptoms are unilateral, which may call the physician's attention concerning the diagnosis of neoplasia^12^.

As far as staging is concerned, preoperative staging based on Krouse's classification, our findings are in agreement with the literature^12^^,^^15^^,^^16^, with a predominance of stage T3 in our study (52.9%). Another finding deserving attention is the higher rate of malignization (17.6% in our study) vis-à-vis the rate mentioned in the literature (5%-15%)^17^. Such fact may be attributed to the sample size as to referral of patients coming from other services.

After the first non-endoscopic approaches showed unacceptable recurrence rates, ranging between 20% all the way to 100%, they ended up being abandoned in favor of external approaches, which had been traditionally considered the gold standard for the surgical treatment of inverted papillomas, since they provide the surgeon with good tumor exposure and satisfactory success rates in the postoperative. However, they were associated with significant intra and postoperative morbidity and longer times for recovery^17^. The development of the endoscope provided a series of advantages in comparison with the external approach, such as better visualization, better lighting and magnification of the operative field, including an angled view, which was not possible with the microscope. It provides for a better resection of viable tissue - not compromised by the tumor, causing less crust formation, less bleeding and less postoperative pain. After proving the efficacy of the endoscopic approach, the traditional approaches ended up losing their usefulness as treatment of choice for the inverted papilloma^4^^,^^9^^,^^17^, ^18^, ^19^, ^20^.

In our study, all the patients classified as T2 were submitted to the endoscopic approach only. Of the nine patients who had stage T3 tumor, six were submitted to the endoscopic approach only, two to the combined approach and one to the external approach only. With this, we used the endoscopic approach only in 64% of the cases. These results suggest the consolidation of using the endoscope for the surgical treatment of the inverted papilloma, not only in less advanced stages as initially proposed, but also in more advanced stages. It is interesting to notice that the resection by piecemeal of the inverted papilloma, such as it is carried out in the endoscopic approach, does not seem to compromise control and recurrence rates when compared to open resection in a single block^17^.

One possible limitation of the endoscopic approach alone, is the access to lesions located in areas such as the frontal sinus or the anterior and inferior walls of the maxillary sinus^12^. However, in a recent retrospective analysis involving 212 patients, only those lesions with extensive frontal and/or supraorbital involvement had to be submitted to a combined approach. Even lesions involving the orbit or intracranial tissues could be treated through the endoscopic approach alone^10^.

Our recurrence rate was 23.5%, which is in agreement with the rates published in the literature^21^ (varying between 20% and 47% in some series^18^^,^^21^). Notwithstanding, when we assess the endoscopic approach only, the recurrence rate was lower −18.1%. This recurrence rate reduction in the endoscopic approach only, when compared to the open procedure is in agreement with papers recently published in the international literature – 14%^12^^,^^22^ on the first *vs*. 25% on the second^22^). However, there are very few papers describing the rates of recurrence vis-à-vis tumor stage, and when they are reported in function of tumor size and staging, an association between more advanced stages and a greater incidence of recurrence has yet to be better clarified^4^^,^^9^^,^^13^. In our study, we did not find a statistically significant correlation between recurrence and a higher tumor stage or malignization.

In a recent literature review, with 63 series of cases and an adequate methodology, with a total of 3,058 patients)^22^, the authors identified 7.1% of synchronous carcinoma. The malignancy rate was 3.6%, and the mean time for such transformation was 52 months. The mean recurrence rates were 12.8% concerning the endoscopic access and 17% for the open procedure, increasing to 34.2% for limited resections^22^.

In relation to postoperative complications, they happened to 17.6% of the patients in our study (septal perforation, oroantral fistulas and synechia, each one in different patients in the sample). In the literature, the rates vary according to the surgical approach - reaching up to 42% in some papers^18^, and the most common complications were: epiphora, facial pain and nasal crusts^2^^,^^18^^,^^23^.

And finally, recent meta-analysis studies have shown lower recurrence rates associated with the endoscopic treatment alone when compared to the external approach (12 % *vs*. 20%)^15^^,^^22^, which supports the exclusive endoscopic treatment for inverted papillomas, even in advanced stages (when there is no extensive involvement of the frontal sinus and/or supraorbital involvement^10^), yielding lower postoperative recurrence rates and complications associated with the procedure - which was seen in the retrospective of our study.

## CONCLUSION

We described the profile of patients with inverted papilloma submitted to surgical treatment in our institution. Our study corroborates data from the national and international literature, proving the efficacy and safety of the endoscopic approach in the surgical treatment of inverted papillomas in more advanced stages.

## References

[1] Ward N. (1854). A mirror of the practice of medicine and surgery in the hospitals of London: London Hospital.: Follicular tumour involving the nasal bones, nasal processes of superior maxillary bone, and the septum of the nose; removal; death from pneumonia; autopsy. Lancet..

[2] Durucu C, Baglam T, Karatas E, Mumbuc S, Kanlikama M. (2009). Surgical treatment of inverted papilloma. J Craniofac Surg..

[3] Krouse JH. (2000). Development of a staging system for inverted papilloma. Laryngoscope..

[4] Han JK, Smith TL, Loehrl T, Toohill RJ, Smith MM. (2001). An evolution in the management of sinonasal inverted papilloma. Laryngoscope..

[5] Brandwien M, Steinberg B, Thung S, Biller H, Dilorenzo T, Galli R. (1989). Human papillomavirus 6/11 and 16/18 in Schneiderian inverted papillomas. In situ hybridization with humas papillomavirus RNA probes. Cancer..

[6] Lawson W, Schlecht NF, Brandwein-Gensler M. (2008). The role of human papillomavirus in the pathogenesis of Schneiderian inverted papillomas: an analytic overview of the evidence. Head Neck Pathol..

[7] Lawson W, Patel ZM. (2009). The evolution of management for inverted papilloma: an analysis of 200 cases. Otolaryngol Head Neck Surg..

[8] Cannady SB, Batra PS, Sautter NB, Roh HJ, Citardi MJ. (2007). New staging system for sinonasal inverted papilloma in the endoscopic era. Laryngoscope..

[9] Krouse JH. (2001). Endoscopic treatment of inverted papilloma: safety and efficacy. Am J Otolaryngol..

[10] Lombardi D, Tomenzoli D, Buttà L, Bizzoni A, Farina D, Sberze F (2011). Limitations and complications of endoscopic surgery for treatment for sinonasal inverted papilloma: a reassessment after 212 cases. Head Neck..

[11] Lian F, Juan H. (2012). Different endoscopic strategies in the management of recurrent sinonasal inverted papilloma. J Craniofac Surg..

[12] Díaz Molina JP, Llorente Pendas JL, Rodrigo Tapia JP, Alvarez Marcos C, Obeso Agüera S, Suárez Nieto C. (2009). Inverted sinonasal papillomas. Review of 61 cases. Acta Otorrinolaringol Esp..

[13] Tomenzoli D, Castelnuovo P, Pagella F, Berlucchi M, Pianta L, Delù G (2004). Different endoscopic surgical strategies in the management of inverted papilloma of sinonasal tract: experience with 47 patients. Laryngoscope..

[14] Salomone R, Matsuyama C, Giannotti Filho O, De Alvarenga ML, Martinez Neto EM, Chaves AG. (2008). Bilateral inverted papilloma: case report and literature review. Braz J Otorhinolaryngol..

[15] Constantino GTM, Abdo TT, Romano FR, Voegels RL, Butugan O. (2007). The role of endoscopic surgery in the treatment of nasal inverted papilloma. Braz J Otorhinolaryngol..

[16] Souza LA, Verde RCL, Lessa MM, Lessa HA, Lima CMF. (2010). Endoscopic treatment of sinonasal papilloma: a retrospective clinical study. Int Arch Otorhinolaryngol..

[17] Busquets JM, Hwang PH. (2006). Endoscopic resection of sinonasal inverted papilloma: a meta-analysis. Otolaryngol Head Neck Surg..

[18] Sadeghi N, Al-Dhahri S, Manoukian JJ. (2003). Transnasal endoscopic medial ma-xillectomy for inverting papilloma. Laryngoscope..

[19] Baruah P, Deka RC. (2003). Endoscopic management of inverted papillomas of the nose and paranasal sinuses. Ear Nose Throat J..

[20] Kraft M, Simmen D, Kaufmann T, Holzmann D. (2003). Long-term results of endonasal sinus surgery in sinonasal papillomas. Laryngoscope..

[21] Kim WS, Hyun DW, Kim CH, Yoon JH. (2010). Treatment outcomes of sinonasal inverted papillomas according to surgical approaches. Acta Otolaryngol..

[22] Mirza S, Bradley PJ, Acharya A, Stacey M, Jones NS. (2007). Sinonasal inverted papillomas: recurrence, and synchronous and metachronous malignancy. J Laryngol Otol..

[23] Sousa AMA, Vicenti AB, Speck Filho J, Cahali MB. (2012). Retrospective analysis of 26 cases of inverted nasal papillomas. Braz J Otorhinolaryngol..

